# The human urinary microbiome; bacterial DNA in voided urine of asymptomatic adults

**DOI:** 10.3389/fcimb.2013.00041

**Published:** 2013-08-15

**Authors:** Debbie A. Lewis, Richard Brown, Jon Williams, Paul White, S. Kim Jacobson, Julian R. Marchesi, Marcus J. Drake

**Affiliations:** ^1^Department of Engineering Design and Mathematics, University of the West of EnglandBristol, UK; ^2^Bristol Urological Institute, Southmead HospitalBristol, UK; ^3^School of Biosciences, Cardiff UniversityCardiff, UK; ^4^North Bristol NHS Trust, Southmead HospitalBristol, UK; ^5^Department of Medical Microbiology, North Bristol NHS Trust, Southmead HospitalBristol, UK; ^6^School of Clinical Sciences, University of BristolBristol, UK

**Keywords:** bladder microbiome, pyrosequencing, urinary microbiome, bladder disease, microbiological methods

## Abstract

The urinary microbiome of healthy individuals and the way it alters with ageing have not been characterized and may influence disease processes. Conventional microbiological methods have limited scope to capture the full spectrum of urinary bacterial species. We studied the urinary microbiota from a population of healthy individuals, ranging from 26 to 90 years of age, by amplification of the 16S rRNA gene, with resulting amplicons analyzed by 454 pyrosequencing. Mid-stream urine (MSU) was collected by the “clean-catch” method. Quantitative PCR of 16S rRNA genes in urine samples, allowed relative enumeration of the bacterial loads. Analysis of the samples indicates that females had a more heterogeneous mix of bacterial genera compared to the male samples and generally had representative members of the phyla Actinobacteria and Bacteroidetes. Analysis of the data leads us to conclude that a “core” urinary microbiome could potentially exist, when samples are grouped by age with fluctuation in abundance between age groups. The study also revealed age-specific genera Jonquetella, Parvimonas, Proteiniphilum, and Saccharofermentans. In conclusion, conventional microbiological methods are inadequate to fully identify around two-thirds of the bacteria identified in this study. Whilst this proof-of-principle study has limitations due to the sample size, the discoveries evident in this sample data are strongly suggestive that a larger study on the urinary microbiome should be encouraged and that the identification of specific genera at particular ages may be relevant to pathogenesis of clinical conditions.

## Introduction

The term “microbiome” refers to all microbiota in a defined microbial community (Dave et al., 2012). With molecular tools now developed to assess the composition and diversity of particular microbiomes far more accurately, and independently of culture methods, potential insights into the affiliation between humans and their associated microbiota in healthy and disease can be obtained. Bacteria constitute 90% of all cells in the human body, and this symbiotic relationship is crucial in maintaining health and for proper development of the host (Proctor, 2011). For example, maintaining a vaginal environment that is dominated by *Lactobacillus* species is associated with healthy pregnancy outcomes, lack of vaginal symptoms and reduced risk for acquiring several sexually transmitted pathogens (Marrazzo, 2011). In addition, studies of the gut have demonstrated a variety of functions for the resident microbiota in maintaining the host's health, including metabolic and trophic functions, as well as providing a protective barrier against pathogens (Guarner and Malagelada, 2003; Chervonsky, 2012).

The bladder was notably not included within the Human Microbiome Project. Historically urine has been considered sterile until reaching the urethra in healthy individuals, hence lacking in an associated microbiota (Fouts et al., 2012). However, urine (as a reflection of the bladder microbiota) from healthy individuals does contain extensive numbers of bacteria, which are not routinely cultivated by clinical microbiology laboratories, but can be identified by 16S rRNA gene sequencing (Nelson et al., 2010; Siddiqui et al., 2011; Wolfe et al., 2012).

The intestinal microbiota of healthy humans progressively develops in complexity from birth until adulthood, where a stable microbiome is established for the majority of an individual's adult life (Arumugam et al., 2011; Jalanka-Tuovinen et al., 2011; Durbán et al., 2012). Yet with aging, physiological changes (e.g., changes in diet, lifestyle, immune system function) induced by the ageing process will likely affect the abundance of an individual's bacteria at every bodily niche (Biagi et al., 2012). Therefore, as for the intestinal microbiota, it is important to understand how the ageing process shapes the microbiota within the healthy, ageing bladder as a platform for future studies (O'Toole, 2012).

The aim of this study was to capture the total urinary microbiota from a sample of healthy individuals of various ages by amplification of the 16S rRNA gene (surrogate marker for the presence of bacteria) with resulting amplicons analyzed by the 454 pyrosequencing high-density system. While bladder biopsies or suprapubic aspirates would provide the best quality material for capturing the bladder microbiota, this was not feasible for a pragmatic study in a healthy population (Wolfe et al., 2012). Accordingly, clean-catch mid-stream urine (MSU) samples were employed to characterize the urinary microbiota, as a reflection of the bladder microbiota. To enumerate the amount of bacteria within each sample and to discriminate contaminants as those that fall below a certain level in a comparable manner to standard urine microbiology tests, DNA within each sample was quantified by qPCR and compared to a known number of operon copies/ml of a urine sample spiked with *Escherichia coli* also quantified by qPCR. This strategy allowed us to relate the relative abundance of bacteria between donated samples and provide more informative data.

## Materials and methods

### Sample collection

Participants were recruited from people attending a secondary care urology clinic and informed consent was obtained from all. The UK South West Central National Research Ethics Service gave ethical approval for the study (NRES reference number 09/H0102/68). The inclusion criteria were as follows: no lower urinary tract symptoms, no history of urinary tract infection (cystitis, pyelonephritis, prostatitis) in the preceding year and no recent use (>1 month) of antibiotics for any indication. Volunteers were instructed to provide a clean-catch, mid-stream voided urine sample into a sterile container. Samples were immediately tested with a urinary dipstick to determine if they were positive for nitrites (Siemens Multistix 8 SG). The samples were anonymized in the order they were received by a local coding system (e.g., UWE01, UWE02 etc.), stored at 4°C and cultured within 4 h of sampling. An aliquot of the samples was frozen at −20°C for subsequent DNA extraction also.

### Extraction of DNA from urine and quality check

DNA was extracted by bead beating in a lysis buffer containing detergent followed by alcohol precipitation. A co-precipitant (sterile linear polyacrylamide, LPA) was included. All buffers were made up from 18.2 MΩ water and autoclaved. Blank extractions were used to confirm zero 16S rRNA gene background contamination in the reagents.

Urine samples (2 ml) were sedimented (2600 g, 5 min) and the pellet washed once in PBS. The pellet was resuspended in 850 μl lysis buffer (3% w/v sodium dodecyl sulphate in 50 mM tris, 5 mM EDTA, pH 8.0, 10 μg/ml RNase A) and transferred to a sterile bead beating tube containing 0.5 g 0.1 mm sterile glass beads. The sample was agitated at 6 m/s for 2 × 45 s. After centrifugation (16000 g, 10 min) 500 μl supernatant was transferred to a fresh tube and 350 μl 5 M ammonium acetate was added and the tubes held on ice for 5 min and centrifuged (16000 g, 5 min). The supernatant was held on ice for a further 5 min and centrifuged (16000 g, 5 min). This was followed by sequentially adding to the supernatant 10 μl 0.5% LPA and 850 μl iso-propyl alcohol. The sample was held at room temperature for 5 min and centrifuged (16000 g, 5 min). The pellet was washed in 500 μl 70% ethanol and re-dissolved in 400 μl TE buffer (10 mM Tris; HCl 1 mM EDTA pH 8.0). To this was added 1 ml 100% ethanol and after 5 min at room temperature the sample was centrifuged (16000 g, 5 min). The pellet was washed in 500 μl 70% ethanol, air dried and dissolved in 50 μl TE buffer. DNA extracts were stored at −80°C.

DNA was confirmed to be of sufficient quality for downstream PCR applications by 16S rRNA PCR by amplifying the V1-V3 hypervariable region of the bacterial 16S rRNA gene using primers 63F (5′ CAGGCCTAACACATGCAAGTC 3′) and 517R (5′ AGGCCTAACACATGCAAGTC 3′). All PCR amplifications were conducted in 50 μl volume containing 5 μl of DNA (10 to <1 ng per reaction depending on sample yield) according to manufacturers of the DNA polymerase (Moltaq 16S, Molzym, Bremen, Germany). The kit was supplied as a 2.5 × mastermix which contains dNTPS, Taq polymerase and MgCl2 (exact content is proprietary). An automated thermal cycler (BioRad, Hemel Hempstead, UK) was used for PCR amplification which was programmed for an initial denaturation of 94°C for 10 min, 40 cycles of denaturation (94°C for 30 s), annealing (60°C for 60 s) and extension (72°C for 120 s) and a final extension of (72°C for 10 min) as per manufacturer's instructions. The samples were verified on a 1.5% w/v agarose gel.

### Quantification of sample bacterial load by quantitative PCR (qPCR) of 16S rRNA DNA

Sample bacterial load was quantified by 16S rRNA qPCR of the urine DNA extracts (Mastermix 16S, Molzym, Bremen, Germany). The kit reagent was supplied as a 2.5 × mastermix which contained dNTPs, primers (343F 5′ TCCTACGGGAGGCAGCAGT 3′ and 809R 5′ GGACTACCAGGGTATCTAATCCTGTT 3′), Taq polymerase and SYBR fluorescent dye in a PCR buffer (exact content is proprietary). A volume of 15 μl of this secondary mix was then added to 10 μl of a 1/10 dilution of respective urine DNA extract/*E. coli* DNA calibrator. The supplied Taq was non hot-start; this necessitated assay set-up on ice, and the use of the PCR cycler plate to be preheated (95°C) in order to minimize primer-dimer formation (verified by melt curve analysis). The amount of urine extract DNA template was 20 to <1 ng/reaction (it was not possible to accurately quantify the amount of DNA in all extracts since some concentrations were <1 ng/μl). The total urine DNA extract volume was 50 μl which originated from 2 ml urine, and 10 μl of a 1/10 dilution of extract was added. The qPCR was calibrated using DNA obtained from an enumerated *E. coli* liquid culture (OD 600 nm of 1.5) extracted by the urine DNA protocol.

In our hands linearity of the enumerated *E. coli* DNA extract was observed over 6 dilution decades from an initial 200 ng/reaction to 200 fg/reaction (equivalent enumerated culture 7 × 10^6^ to 70 cfu per reaction, 4.9 × 10^7^ to 490 operon copies per reaction using a copy value of 7 operons per CFU; Klappenbach et al., 2001). This yielded a urine assay range (obtained by calculation from extraction factorization) of 1.75 × 10^8^ to 1.75 × 10^3^ *E. coli* cfu/ml which is 1.25 × 10^9^ to 1.25 × 10^4^ operon copies/ml equivalent. The qPCR assay was susceptible to primer-dimer interference. Optimization experiments indicated that under the employed conditions a false signal originated in the negative sample (blank) and low copy sample extracts at 30 cycles. This yielded a practical detection limit of 10^4^ cfu/ml or 7 × 10^4^ operon copies/ml.

### Sequencing and analysis

FLX-titanium amplicon pyrosequencing (bTEFAP) of the VI-V3 regions of the 16S rRNA gene was performed by Research and Testing Laboratory (Lubbock, Tx, USA) and the tagged reads were denoized and chimeras removed prior to analysis in the host institution (Edgar et al., 2011). Once the reads were transferred they were analyzed by means of the bioinformatic software Mothur (http://www.mothur.org: version 1.28.1) using absolute numbers in each sample to minimize any artifacts which might be introduced due to re-sampling (Schloss et al., 2009). The reads were mapped to the Ribosomal Database Project (RDP) taxonomy by Mothur using a dissimilarity cutoff value of 0.03 and only operational taxonomic units (OTUs) with a minimum of 10 reads in any sample were used. The unique OTUs from this pipeline were used in the R statistical environment to determine clusters and heatmaps. The number of clusters was determined using partitioning around medoids (PAMK) in the package FPC in R. The 16S rRNA gene sequences are available for download from the European Nucleotide Archive (ENA) under accession number PRJEB4256.

## Results

### Sample collection

MSU samples were collected by the clean-catch method from male (*n* = 6; age range 39–83 years of age) and female (*n* = 10; age range 26–90 years of age) donors. DNA extractions and PCR analysis of V1–V3 hypervariable region using 63F and 517R primers was performed to ensure samples were sufficient to be amplifiable by downstream PCR methods. Samples were confirmed to be of good quality DNA and were sent to the Research and Testing Laboratories (Lubbock, Texas) for 454 pyrosequencing with returned results as shown in Table 1.

**Table 1:** **Table listing the sex and age of each sample including the “equivalent” number of rRNA operon copies/ml**.

**Sample ID**	**Age**	**Sex**	**Positive for nitrites^a^**	**rRNA operon copies/ml of urine equivalent^b^**	**Total no. of genera present in sample**
UWE01	78	Female	No	3.71 × 10^6^	22
UWE02	47	Female	No	5.5 × 10^6^	30
UWE04	39	Male	Yes	8.4 × 10^8^	3
UWE06	55	Female	No	BDL	9
UWE15	71	Male	Yes	1.3 × 10^6^	51
UWE18	26	Female	No	1.75 × 10^7^	20
UWE19	67	Male	Yes	5.5 × 10^6^	4
UWE20	79	Female	No	1.3 × 10^6^	12
UWE22	40	Male	No	9.8 × 10^6^	2
UWE23	69	Male	No	2.2 × 10^7^	8
UWE24	62	Female	No	BDL	27
UWE30	83	Male	No	3.4 × 10^5^	1
UWE32	36	Female	Yes	2.4 × 10^5^	36
UWE34	58	Female	No	BDL	16
UWE44	90	Female	No	2.5 × 10^6^	31
UWE47	78	Female	Yes	4.6 × 10^5^	6

Included also is the total no. of genera present in each sample.

a As determined by urinary dipstick.

b As determined by qPCR extrapolated from known numbers of E. coli; BDL, Below detection limit of method; n/a, not applicable.

### Taxonomic assignment of the sequences

**Figure 1:**
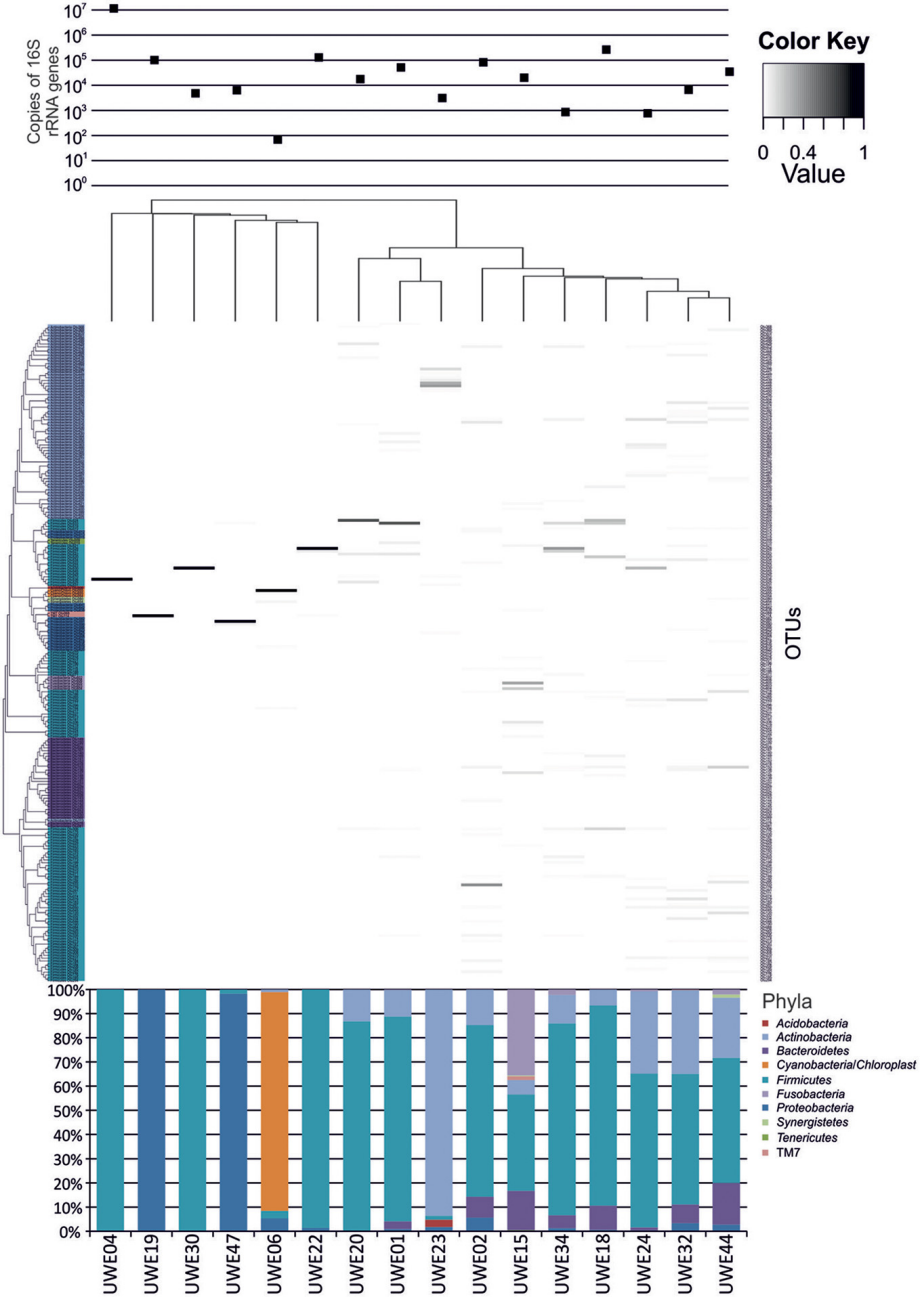
**Heatmap showing the relative abundance of the OTUs per sample.** The **top panel** presents the qPCR values for each sample while the **bottom panel** shows the percentage distribution of the OTUs' phyla for each sample.

**Figure A1:**
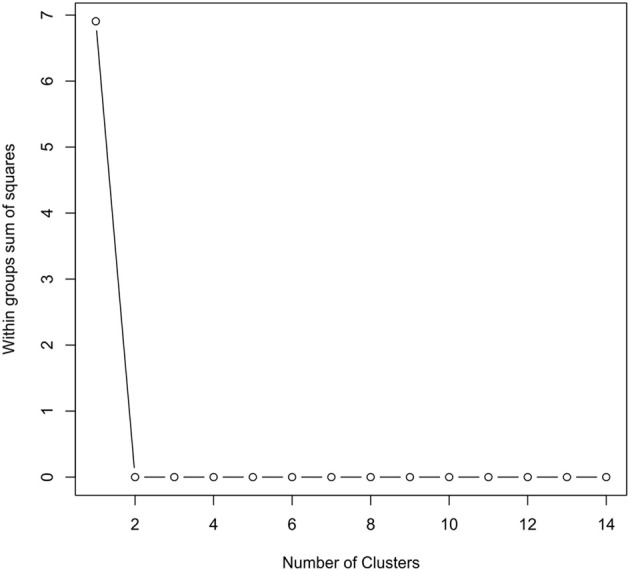
**Graphical view of the results of the partitioning around mediods (PAMK in R) used to determine the number of clusters in the urinary microbiome datasets.** In this instance two clusters were identified as being the optimal solution.

The 16S rRNA gene sequences were classified using the bioinformatics software Mothur and a dissimilarity cut-off of 0.03, into 663 OTUs. Upon further analysis, according to criteria stipulated in the methods, this was reduced to 234 OTUs and came from 10 phyla, 17 classes, 27 orders, 56 families, and 94 genera (Table A1). The microbiota of the 16 samples clustered into two groups as determined by the partitioning around mediods method (PAMK in R); those that were dominated by one phylum and those with more than one phylum (Figure A1). Of the monophyletic samples, three (UWE04, UWE22, and UWE30) were dominated by OTUs which clustered in the *Firmicutes*, while two (UWE19 and UWE47) were dominated by OTUs from the *Proteobacteria*. In the samples in which one phylum was present there was commonly only one OTU represented from that phylum; OTU525: genus *Staphylococcus* (in sample UWE04), OTU527: genus *Lactobacillus* (UWE22), OTU545: genus *Aerococcus* (UWE30), OTU582: genus *Pseudomonas* (UWE19), and OTU590: genus *Enterobacter* (UWE47) (Figure 1). The remaining samples had on average 5 phyla (range 2–8; Table A2).

### Enumeration of 16S rRNA genes by qPCR

To enable quantitative comparison between urine samples a qPCR assay of the 16S rRNA gene was done and compared to a known enumerated *E. coli* sample. Due to the inter species variation in rRNA operons per genome it was decided to express the urine bacterial load arbitrarily as operon copy/ml equivalent, using a copy value for *E. coli* of 7 operons per CFU (Klappenbach et al., 2001).

Table 1 shows the “equivalent” number of rRNA operon copies/ml of urine per patient and also the total number of different genera which were present within each sample. In 6 of the samples (5 female—UWE01, UWE02, UWE18, UWE20, and UWE44; and 1 male—UWE 22) the results of the urinary dipstick for the detection of nitrites as an indication of the presence of bacteria was negative but results from the qPCR suggest the bacterial count would likely be in numbers far greater than 1 × 10^5^ cfu/ equivalent based on the copy value of *E. coli* of 7 operons per CFU. Equally two female samples were positive to nitrites (UWE32 and UWE44) however bacterial counts would likely be less than 1 × 10^5^ cfu/ml equivalent, as determined by qPCR. Overall therefore 50% of samples could have been potentially misclassified based on the urinary dipstick analysis alone.

### Diversity of bacteria within each gender as individuals

When considering the 16 samples collectively, the general trend is a more heterogeneous mix of bacterial genera in the female samples (range 6–36, average 21, median 21) than the male (range 1–8 plus one sample of 51, average 11.5, median 3.5) (Table 1; Figure 1). Analysis using the non-parametric Mann Whitney test indicates that the distribution of total genera is sex dependent (*Z* = 2.061, *n*1 = 10, *n*2 = 6, *p* = 0.042, two-sided). Three quarters of samples have greater than 50% of the total abundance of bacteria belonging to the phylum *Firmicutes* and this was true for both male and female samples. Female samples also generally had representative members of the phyla *Actinobacteria* and *Bacteroidetes*, which were generally absent from the male samples.

### Diversity of bacteria by age as individuals

**Figure 2:**
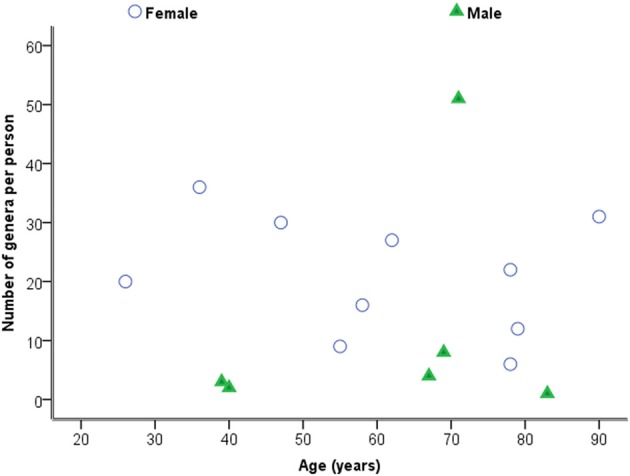
**Plot of number of genera against age by sex**.

Considering samples individually in the first instance, Figure 2 is a plot of the total number of genera identified per person against age. Statistical analysis using Spearman's correlation coefficient indicates that the number of genera and age are not significantly correlated in the sample (*r* = −0.029, *p* = 0.914, two-sided).

**Figure 3:**
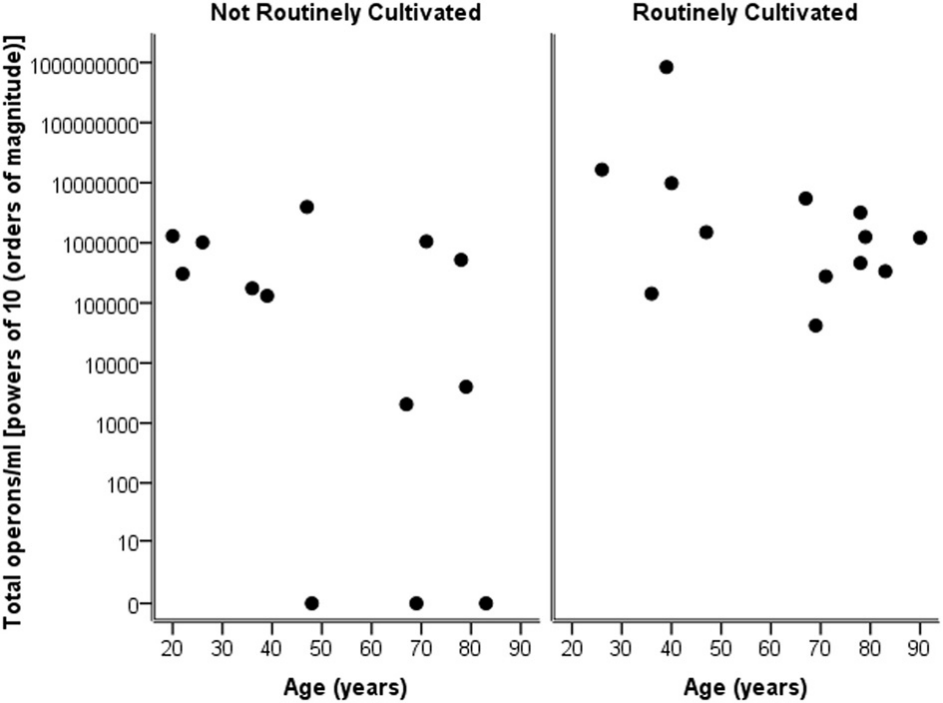
**Plot of total operons/ml per person (powers of 10—order of magnitude) for each genus that is cultivated routinely by standard microbiological testing, and those not routinely cultivated (including those not individually identified in routine culture)**.

Considering the number of operons/ml as determined by qPCR for both routinely cultivated and not routinely cultivated individually, it can be seen in Figure 3 that the “total” number of bacteria is similar across the age for both groups. Note “not routinely cultivated” also includes those not reported individually by standard methods; please see Tables A3, A4 for more details on groupings. Further analysis using Spearman's rank correlation coefficient indicates that the sample relationship between operons/ml and age does not achieve statistical significance for genera not routinely cultivated (*r* = −0.464, *p* = 0.110, two-sided) or for routinely cultivated genera (*r* = −0.391, *p* = 0.187, two-sided).

**Figure 4:**
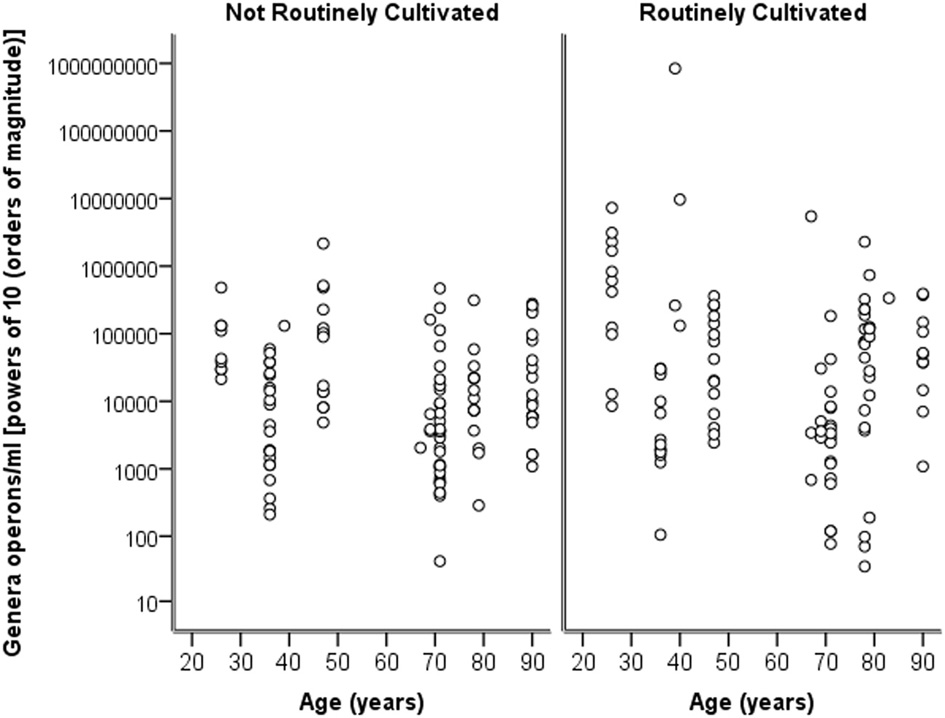
**A plot of the genus specific count (operons/ml) on a logarithmic base ten scale against age for routinely cultivated and not routinely cultivated bacteria (including those not individually identified in routine culture)**.

Breaking down the “total” number of bacteria per participant into the “total” numbers of bacteria for each genera against age, it can again be seen in Figure 4 that numbers are highly similar across the ages and for both routinely cultivated and not routinely cultivated bacteria.

### Diversity of bacteria by age when grouped

When samples are grouped into the following age ranges: 20–49 years, 50–69 years, and 70 plus years, irrespective of gender it is notable that the following genera appeared exclusive to the 70 plus age group, namely *Jonquetella, Parvimonas, Proteiniphilum*, and *Saccharofermentans*. Analysis using the non-parametric Mann Whitney test indicates that the distribution of the frequencies of this cluster of genera is age dependent (*Z* = 3.873, *n*1 = 6, *n*2 = 10, *p* < 0.001, two-sided).

### Diversity of bacteria by age and gender when grouped

The sixteen samples were grouped within the following age ranges: 20–49 years, 50–69 years, and 70 plus years.

For females aged 20–49 (*n* = 3) the number of different genera identified was 48, with 30 of these having a predicted operons/ml of greater than the detection limit by qPCR (7 × 10^4^ operons/ml equivalent) (Table 2). The average total operons/ml for all bacteria for the females within the age group 20–49 (*n* = 3) was 1.3 × 10^5^. For females within the age group 50 to 69+ (*n* = 3) the number of genera present was 36. It was not however possible to predict operons/ml within this group for any of the samples, as the readings were below the detection limit of the qPCR method. Nonetheless, it does suggest that the bacterial counts were much lower than the other two groups who reached at least the detection limit. In the 70+ age group the number of different genera identified was 43 (*n* = 4). The average total operons/ml for all bacteria for the females in this age group (70+) was 3.3 × 10^4^. Therefore in the 70+ female age group, there were approximately 75% fewer bacteria (operons/ml) than in the age group 20–49. Notably, the age groups 20–49 and 70+ had in common 17 genera that were present in both groups at numbers greater than the predicted 7 × 10^3^ operons/ml (Table 2). A further 6 genera were common within these groups but detectable in greater numbers (>7 × 10^3^ operons/ml) in the age group 20–49, than in the 70+ (Table 2).

**Table 2:** **Table detailing the genera identified within each defined age group for females only**.

**All ages (*n* = 23)**	**Age 20–49 (*n* = 13)**	**Age 50–69 (*n* = 9)**	**Age 70+ (*n* = 11)**	**Age 20–49 and 50–69 (*n* = 4)**	**Age 50–69 and 70+ (*n* = 1)**	**Age 20–49 and 70+ (*n* = 9)**
*Actinobaculum*	***Azospira***	*Brevibacterium*	*Actinomyces*	*Aerococcus*	*Enterobacter*	***Anaerovorax***
*Anaerococcus*	***Butyricicoccus***	***Catonella***	*Arthrobacter*	*Arcanobacterium*		***Flavonifractor***
*Anaerosphaera*	***Coriobacterium***	***Caulobacter***	***Gulosibacter***	***Brooklawnia***		***Gallicola***
*Atopobium*	***Friedmanniella***	***Methylovirgula***	***Jonquetella***	***Fastidiosipila***		***Helcococcus***
*Campylobacter*	*Gardnerella*	***Pelomonas***	***Lachnospiracea_incertae_sedis***			***Howardella***
*Corynebacterium*	***Microvirgula***	*Peptostreptococcus*	***Modestobacter***			*Peptococcus*
*Dialister*	*Neisseria*	***Sneathia***	***Oligella***			***Soehngenia***
*Enterobacter*	*Paraprevotella*	***Streptophyta***	***Parvimonas***			*Staphylococcus*
*Enterocococcus*	***Rhodopila***	***Thermoleophilum***	***Proteiniphilum***			*Stenotrophomonas*
*Facklamia*	***Sutterella***		*Rhodococcus*			
*Finegoldia*	***Tepidimonas***		***Saccharofermentans***			
*Fusobacterium*	***Tessaracoccus***					
*Lactobacillus*	***TM7_genera_incertae_sedis***					
*Mobiluncus*						
*Murdochiella*						
*Negativicoccus*						
*Peptoniphilus*						
*Porphyromonas*						
*Prevotella*						
*Propionimicrobium*						
*Sporanaerobacter*						
*Streptococcus*						
*Varibaculum*						

Those highlighted in bold are not routinely cultivated and/or reported individually by standard methods described the UK Health Protection Agency in routine investigations of urine (Health Protection Agency, 2012).

The males presented a different picture, in that the numbers of genera present increased through the age groups (Table 3). However, despite the increase in number of genera within the male age group 70+, the average total number of bacteria was lower than the younger age groups with a predicted 4.9 × 10^7^ operons/ml in the age group 20–49, compared with 9 × 10^4^ operons/ml in the age group 50–69 and 1 × 10^5^ operons/ml in the 70+ age group. Analysis using the Kruskal-Wallis test shows that these observed sample difference do not achieve statistical significance (*H* = 2.593, *df* = 2, *p* = 0.273).

**Table 3:** **Table detailing the genera identified within each defined age group for males only**.

**All ages (*n* = 1)**	**Age 70+ (*n* = 48)**		**Age 20–49 and 50–69 (*n* = 1)**
*Staphylococcus*	*Aerococcus*	***Kocuria***	*Pseudomonas*
	***Aminobacterium***	***Lactonifactor***	
	*Anaerococcus*	***Marixanthomonas***	
	***Anaerophaga***	***Megasphaera***	
	***Anaerosphaera***	***Microvirgula***	
	***Anaerotruncus***	*Mobiluncus*	
	***Atopobium***	***Murdochiella***	
	***Atopostipes***	***Mycoplasma***	
	***Azospira***	***Parvimonas***	**Age 20 and 70+ (*n* = 2)**
	***Butyricicoccus***	*Peptococcus*	***Actinobaculum***
	*Campylobacter*	***Peptoniphilus***	*Lactobacillus*
	***Catonella***	*Peptostreptococcus*	
	*Corynebacterium*	*Porphyromonas*	
	***Dialister***	*Prevotella*	
	*Eubacterium*	***Proteiniphilum***	
	***Filifactor***	***Pseudoramibacter***	
	*Finegoldia*	***Rikenella***	
	*Fusobacterium*	***Saccharofermentans***	
	*Gardnerella*	***Sediminitomix***	
	*Gemella*	***Sneathia***	
	***Gordonibacter***	***Soehngenia***	

Those highlighted in bold are not routinely cultivated and/ or reported individually by standard methods described the UK Health Protection Agency in routine investigations of urine (Health Protection Agency, 2012).

## Discussion

Understanding how the human microbiota develops and changes with ageing is essential for future studies investigating the effects of changes in the microbiota, and implications for maintaining a healthy host and whether any disease state results. Physiological changes that are induced by the ageing process, age-related events (for example morbidity, medication and lifestyle) and the reduction of functionality of the immune system or immunosenescence will inevitably modify the composition of the microbiota throughout the human body (Gruver et al., 2007). This change has been clearly demonstrated in the gut microbiota, sampling faeces, and the evidence provided here suggests it is likely an analogous situation occurs in the bladder and sampling urine (Tiihonen et al., 2010; Jalanka-Tuovinen et al., 2011; Biagi et al., 2012; O'Toole, 2012; Yatsunenko et al., 2012).

The method of sampling is frequently debated in any study relating the microbiota of the urine (Nelson et al., 2010; Dong et al., 2011; Wolfe et al., 2012). The difficulties in getting a genuine “clean-catch” MSU sample representative of only organisms originating from the bladder is well-recognized and especially so for females, in which the urine has to pass through the distal urethra and/or for the elderly (both male and female), who have physical or other impairments (Franz and Hörl, 1999; Lifshitz and Kramer, 2000; Wilson and Gaido, 2004). Nonetheless, it is less intrusive than alternative approaches, such as bladder tissue biopsy or catheterization, and hence the most pragmatic and ethical approach for larger, longitudinal studies and clinical application and it was therefore our positive intention to collect samples in this way (Wolfe et al., 2012). However this is not withstanding potential urethral or perineal contamination which in a larger study could be considered by determining thresholds (operons/ml) in which to disregard bacteria that fall below this as potential contaminants. Further work could also include taking a urethral swab for comparison to the urine sample to identify with greater clarity what most likely resides truly in the urethra and has simply contaminated the urine sample.

Samples were collected from participants ranging in age from 26 to 90 years of age from both genders. Whilst these participants had no lower urinary tract symptoms and no history of urinary tract infection in the preceding year it is worth noting that the participants were visiting the hospital as an out-patient for other unspecified treatments and may not therefore be considered representative of the primary care population. Samples were also only included if antibiotics had not been taken for any indication in the preceding month. However a study by Dethlefsen and Relman (2011) suggests it may take greater than 2 months to return to a baseline microbiota or at least an altered stable composition. We also acknowledge that certain groups, for example the elderly are likely to have had repeated antibiotic exposure which will result in the possibility of greater dynamic change to the bladder microbiota in comparison to a different age group. Therefore the comparison of samples within age groups in a larger study will be essential.

Culture-independent molecular PCR methods were used to analyse all samples which included 454 pyrosequencing and quantitative PCR, as routine culture methods would not capture the entire urinary microbiota (Imirzalioglu et al., 2008; Wolfe et al., 2012). The sequencing results identified 94 different genera with only 31 likely to be routinely cultivated by standard culture methods. The remaining 63 are either not routinely cultivated or would not be reported individually by routine urine investigations in an NHS microbiology laboratory (Health Protection Agency, 2012). Therefore there is an important discrepancy between what is reported by United Kingdom National Health Service microbiology laboratories and what can actually be detected within urine using molecular methods.

To allow for direct, quantifiable comparisons of the bacterial abundance between each sample, each sample was measured by qPCR. This was to determine the number of copies of 16S rRNA gene against a spiked urine sample with a known number of *Escherichia coli*, also measured by qPCR to determine the number of copies of the 16S rRNA gene. We acknowledge this method has limitations, for example the assumption that all bacteria have the same copy number for the 16S rRNA gene leading to an over- or under-estimation for many bacteria. It does, however, allow us to directly compare the abundance of the different genera between samples and give us an indication at the very least of the number of colony forming units per ml of urine of bacteria.

In agreement with Fouts et al. (2012), the data presented here concurs that the different sexes have significantly different genera of bacteria present in their urine, different numbers of genera and that sequences in the main belong to the phylum *Firmicutes* for both male and female samples, as in the study by Siddiqui et al. (2011). The general lack of representatives from the phyla *Actinobacteria* and *Bacteroidetes* in the male samples within this study also seems typical in comparison to other studies (Nelson et al., 2010; Dong et al., 2011). The current study also describes the first observation of the genera *Soehngenia* detected within a sample originating from a human, in this instance urine, seen in four of the subjects, male (*n* = 1) and female (*n* = 3). The fastidious anaerobic bacteria from this genus would not be detected by standard methods (Parshina et al., 2003).

The correlation of the results of the urinary dipstick as a preliminary indication of bacterial presence of numbers greater than 1 × 10^5^ cfu/ml has again been questioned within this study. Semeniuk and Church (1999) reported that approximately 20% of samples by urinary dipstick analysis would have been sent to the laboratory for microbiology testing and therefore not screened for bacteriuria. In this study, eight out of 16 samples could potentially have been misclassified by urinary dipstick analysis, either being called “negative” when actually positive on qPCR (*n* = 6), or “positive” when actually negative (*n* = 2). Additionally, by quantifying the total number of bacteria by qPCR methods, we have shown that the numbers of bacteria that are routinely cultivated are comparable in amount to the numbers not routinely cultivated and/or identified individually. This raises questions on the precision of methods used in routine microbiological investigation for reporting a total bacterial count. By inaccurately informing clinicians of the presence/absence and quantity of bacteria, there may be serious implications in any treatment plan of patients.

Whilst statistical significance was not achieved, there is marginal evidence that the numbers of genera decrease with age when individually considered, but that the total number of bacteria is independent of age. However the use of relatively small numbers of donors in the context of many different bacteria being identified, affects the ability of the study to make definitive conclusions.

It is clear that the bacterial composition in the urine collected within this study is highly variable regardless of sex or age. This makes it unlikely that a comparable bacterial community between individuals of the same age or gender exists, just as Siddiqui et al. (2011) suggest. Nevertheless, quantifying the samples by qPCR has allowed us directly to compare one sample to another, and thus group samples together according to their age and sex for collated comparisons. Dividing into arbitrary age groups, the data is suggestive of the existence of a “core” bladder microbiome, with variability in the amount of the “core” bacteria and flux of other bacteria with ageing, much like the gut microbiome (Arumugam et al., 2011; Jalanka-Tuovinen et al., 2011). The female samples perhaps best demonstrate this. Across all three age groups, the female samples have in common 23 genera, the “core” microbiome, with each age group additionally having their own distinct subset of genera. There are also a number of genera that occur within two age groups, suggesting a transitional period may occur. Unfortunately the qPCR method for enumerating/predicting bacterial counts was not valid for the samples within the 50–69 age group, as the samples were below the detection limit of the method. However, it is interesting that the age groups 20–49 and the 70+ have 32 genera in common; 17 were present at greater than 10^3^ “*E. coli* equivalent” cfu/ml as determined by qPCR, so highly unlikely to be contaminants. Six of these genera were found in higher numbers in the age group 20–49 than the age group 70+, suggesting that these bacteria may remain present throughout life, but their quantity could be indicative of the age group.

Peters et al. (2009) have argued a link between earlier episodes of UTI in life, with the increasing prevalence of such conditions as interstitial cystitis (IC) and painful bladder syndrome (PBS). The data here could support the hypothesis that bladder colonization with specific genera in early child/adulthood (asymptomatic or symptomatic) might influence propensity to bladder pathology in later life as one factor in the multi-factorial basis of disease pathogenesis.

For both the male and female samples, the average total number of urinary bacteria measured by predicting operons/ml decreased with increasing age when considered in groups. Confirmation of this observation, and mechanisms responsible, would need to be addressed in a more substantial study but one possible reason is varying bacteria load according to sexual activity and therefore contamination of the urine from the urethra (Dong et al., 2011). However, for both the male and female samples within the age group 70+, four different genera unique to this age category were identified, namely bacteria of the genera *Jonquetella, Parvimonas, Proteiniphilum*, and *Saccharofermentans*. These bacteria are strict anaerobes and not routinely cultured, suggesting the possibility of specific bacteria more likely affecting the elderly age-groups but will need to be further explored in a wider study with greater numbers.

This study provides a catalogue of bacterial DNA identified in voided urine from a small cross-sectional sample of healthy adults. It additionally provides data giving an insight into the possibility of a “core” urinary microbiome, (which may potentially fluctuate in abundance with age) and provides data consistent with the concept of there being age and sex specific genera. The microbiological methods in current routine urine assessment are unlikely to identify many of these bacteria and certainly not accurately able to enumerate them, necessitating the need for alternative testing systems to be explored in due course. By quantifying the MSU sample with qPCR against an enumerated *E. coli* sample it has been possible to postulate “contaminant” bacteria as those with very few numbers (<10^3^ cfu/ml or <7 × 10^3^ operons/ml “*E. coli* equivalent”).

In conclusion, the discoveries evident in this sample data suggest there will be considerable scientific interest in a larger, longitudinal study on the urinary microbiome.

### Conflict of interest statement

The authors declare that the research was conducted in the absence of any commercial or financial relationships that could be construed as a potential conflict of interest.

## Appendix

**Table A1:** **The taxonomic groups identified for all the samples at the Phylum, Class, Order, Family, and Genus levels**.

**Phylum 10 Phyla**	**Class 17 Classes**	**Order 27 Orders**	**Family 56 Families**	**Genus 94 Genera**
*Proteobacteria*	*Betaproteobacteria*	*Burkholderiales*	*Burkholderiales_incertae_*	*Rivibacter*
*Acidobacteria*	*Acidobacteria*_Gp10	Gp10	*sedis*	Gp10
*Tenericutes*	*Mollicutes*	*Mycoplasmatales*	*Gp10*	*Mycoplasma*
*Bacteroidetes*	*Epsilonproteobacteria*	*Campylobacterales*	*Mycoplasmataceae*	*Campylobacter*
*Firmicutes*	*Bacteroidia*	*Bacteroidales*	*Campylobacteraceae*	*Paraprevotella*
*Cyanobacteria/Chloroplast*	*Flavobacteria*	*Flavobacteriales*	*Prevotellaceae*	*Rikenella*
*Actinobacteria*	*Clostridia*	*Clostridiales*	*Rikenellaceae*	*Proteiniphilum*
TM7	*Chloroplast*	*Chloroplast*	*Porphyromonadaceae*	*Prevotella*
*Synergistetes*	*Actinobacteria*	*Thermoleophilales*	*Marinilabiaceae*	*Anaerophaga*
*Fusobacteria*	TM7_*genera_incertae_*	TM7_*genera_incertae_*	*Flavobacteriaceae*	*Marixanthomonas*
	*sedis*	*sedis*	*Clostridiales_Incertae Sedis*	*Sporanaerobacter*
	*Bacilli*	*Lactobacillales*	*XI*	*Gallicola*
	*Sphingobacteria*	*Sphingobacteriales*	*Incertae Sedis XI*	*Anaerosphaera*
	*Synergistia*	*Synergistales*	*Chloroplast*	*Streptophyta*
	*Negativicutes*	*Coriobacteriales*	*Thermoleophilaceae*	*Thermoleophilum*
	*Gammaproteobacteria*	*Selenomonadales*	TM7_*genera_incertae_sedis*	TM7_*genera_incertae_sedis*
	*Fusobacteria*	*Actinomycetales*	*Ruminococcaceae*	*Anaerotruncus*
	*Alphaproteobacteria*	*Bifidobacteriales*	*Carnobacteriaceae*	*Murdochiella*
		*Bacillales*	*Lachnospiraceae*	*Atopostipes*
		*Pseudomonadales*	*Flammeovirgaceae*	*Lactonifactor*
		*Enterobacteriales*	*Synergistaceae*	*Butyricicoccus*
		*Xanthomonadales*	*Coriobacteriaceae*	*Sediminitomix*
		*Fusobacteriales*	*Veillonellaceae*	*Aminobacterium*
		*Neisseriales*	*Clostridiales_Incertae Sedis*	*Finegoldia*
		*Rhodocyclales*	*XIII*	*Flavonifractor*
		*Rhodospirillales*	*Peptococcaceae 1*	*Coriobacterium*
		*Rhizobiales*	*Eubacteriaceae*	*Saccharofermentans*
		*Caulobacterales*	*Peptostreptococcaceae*	*Porphyromonas*
			*Actinomycetaceae*	*Anaerococcus*
			*Corynebacteriaceae*	*Peptoniphilus*
			*Propionibacteriaceae*	*Negativicoccus*
			*Micrococcaceae*	*Soehngenia*
			*Bifidobacteriaceae*	*Howardella*
			*Geodermatophilaceae*	*Dialister*
			*Nocardiaceae*	*Anaerovorax*
			*Pseudonocardiaceae*	*Peptococcus*
			*Mycobacteriaceae*	*Fastidiosipila*
			*Nocardioidaceae*	*Megasphaera*
			*Brevibacteriaceae*	*Catonella*
			*Microbacteriaceae*	*Parvimonas*
			*Streptococcaceae*	*Syntrophococcus*
			*Enterococcaceae*	*Eubacterium*
			*Lactobacillaceae*	*Filifactor*
			*Aerococcaceae*	*Pseudoramibacter*
			*Staphylococcaceae*	*Peptostreptococcus*
			*Bacillales_Incertae Sedis XI*	*Helcococcus*
			*Pseudomonadaceae*	*Lachnospiracea_incertae_*
			*Enterobacteriaceae*	*sedis*
			*Xanthomonadaceae*	*Actinobaculum*
			*Fusobacteriaceae*	*Corynebacterium*
			*Leptotrichiaceae*	*Varibaculum*
			*Neisseriaceae*	*Mobiluncus*
			*Sutterellaceae*	*Arcanobacterium*
			*Comamonadaceae*	*Propionimicrobium*
			*Rhodocyclaceae*	*Atopobium*
			*Alcaligenaceae*	*Gordonibacter*
			*Acetobacteraceae*	*Trueperella*
			*Beijerinckiaceae*	*Kocuria*
			*Caulobacteraceae*	*Gardnerella*
				*Actinomyces*
				*Modestobacter*
				*Rhodococcus*
				*Pseudonocardia*
				*Mycobacterium*
				*Gordonia*
				*Nocardioides*
				*Brooklawnia*
				*Brevibacterium*
				*Friedmanniella*
				*Tessaracoccus*
				*Gulosibacter*
				*Arthrobacter*
				*Streptococcus*
				*Enterococcus*
				*Lactobacillus*
				*Facklamia*
				*Staphylococcus*
				*Gemella*
				*Aerococcus*
				*Pseudomonas*
				*Enterobacter*
				*Stenotrophomonas*
				*Fusobacterium*
				*Sneathia*
				*Neisseria*
				*Sutterella*
				*Pelomonas*
				*Azospira*
				*Microvirgula*
				*Tepidimonas*
				*Oligella*
				*Rhodopila*
				*Methylovirgula*
				*Caulobacter*
				*Jonquetella*

**Table A2:** **Percentage of OTUs in each phyla for the 19 samples analyzed**.

	**UWE01**	**UWE02**	**UWE04**	**UWE06**	**UWE15**	**UWE18**	**UWE19**	**UWE20**	**UWE22**	**UWE23**	**UWE24**	**UWE30**	**UWE32**	**UWE34**	**UWE44**	**UWE47**
*Proteobacteria*	0.91	5.63	0	5.31	0.21	0.61	99.89	0	1.34	1.77	0.41	0	3.41	1.28	2.77	98.18
*Acidobacteria*	0	0	0	0	0	0	0	0	0	2.99	0	0	0	0	0	0
*Tenericutes*	0	0	0	0	0.27	0	0	0	0	0	0	0	0	0	0	0
*Bacteroidetes*	3.23	8.67	0	0	16.23	10	0	0	0	0	1.23	0	7.65	5.33	17.17	0
*Firmicutes*	84.66	70.94	99.98	3.15	39.79	82.77	0.08	86.83	98.66	1.66	63.51	100	54.03	79.37	51.73	1.75
*Cyanobacteria/*	0	0	0	90.41	0	0	0.04	0	0	0	0	0	0	0	0	0
*Chloroplast*																
*Actinobacteria*	11	14.76	0.02	1.13	6.04	6.57	0	13.17	0	93.58	34.03	0	34.61	11.72	24.88	0.07
TM7	0	0	0	0	1.60	0	0	0	0	0	0	0	0.29	0	0	0
*Synergistetes*	0	0	0	0	0.27	0	0	0	0	0	0	0	0	0	1.33	0
*Fusobacteria*	0.2	0	0	0	35.58	0.05	0	0	0	0	0.82	0	0	2.3	2.11	0
Number of phyla	5	4	1	4	8	5	1	2	1(2)^a^	4	5	1	5	5	6	1(2)^a^

a These two samples are treated as having one predominant phyla.

**Table A3:** **Table listing all the bacteria identified in this study that are not routinely cultivated or reported individually by standard methods described by the Health Protection Agency in routine investigations of urine (“Health Protection Agency, 2012”)**.

**Genus**	**Aerobe or Anaerobe**	**Gram status**
*Actinobaculum*	Anaerobe	Positive
*Aminobacterium*	Anaerobe	Negative
*Anaerophaga*	Anaerobe	Negative
*Anaerosphaera*	Anaerobe	Positive
*Anaerotruncus*	Anaerobe	Positive
*Anaerovorax*	Anaerobe	Positive
*Atopobium*	Anaerobe	Positive
*Atopostipes*	Anaerobe	Positive
*Azospira*	Anaerobe	Negative
*Brooklawnia*	Anaerobe	Positive
*Butyricicoccus*	Anaerobe	Positive
*Catonella*	Anaerobe	Negative
*Caulobacter*	Aerobe	Negative
*Coriobacterium*	Anaerobe	Positive
*Dialister*	Anaerobe	Negative
*Facklamia*	Anaerobe	Positive
*Fastidiosipila*	Anaerobe	Positive
*Filifactor*	Anaerobe	Positive
*Flavonifractor*	Anaerobe	Positive
*Friedmanniella*	Anaerobe	Positive
*Gallicola*	Anaerobe	Positive
*Gordonibacter*	Anaerobe	Positive
*Gordonia*	Aerobe	Positive
*Gp10*	Uncultured	
*Gulosibacter*	Aerobe	Positive
*Helcococcus*	Aerobe	Positive
*Howardella*	Anaerobe	Positive
*Jonquetella*	Anaerobe	Negative
*Kocuria*	Anaerobe	Positive
*Lachnospiracea_incertae_sedis*	Anaerobe	Positive
*Lactonifactor*	Anaerobe	Positive
*Marixanthomonas*	Aerobe	Negative
*Megasphaera*	Anaerobe	Negative
*Methylovirgula*	Aerobe	Negative
*Microvirgula*	Aerobe	Negative
*Modestobacter*	Aerobe	Positive
*Murdochiella*	Anaerobe	Positive
*Mycoplasma*	Anaerobe	Positive
*Negativicoccus*	Anaerobe	Negative
*Oligella*	Aerobe	Negative
*Parvimonas*	Anaerobe	Positive
*Pelomonas*	Aerobe	Negative
*Peptoniphilus*	Anaerobe	Positive
*Proteiniphilum*	Anaerobe	Negative
*Pseudonocardia*	Anaerobe	Positive
*Pseudoramibacter*	Anaerobe	Positive
*Rhodopila*	Anaerobe	Negative
*Rikenella*	Anaerobe	Negative
*Rivibacter*	Aerobe	Negative
*Saccharofermentans*	Anaerobe	Positive
*Sediminitomix*	Aerobe	Negative
*Sneathia*	Anaerobe	Negative
*Soehngenia*	Anaerobe	Positive
*Sporanaerobacter*	Anaerobe	Positive
*Streptophyta*	Algae	
*Sutterella*	Anaerobe	Negative
*Syntrophococcus*	Anaerobe	Negative
*Tepidimonas*	Aerobe	Negative
*Tessaracoccus*	Anaerobe	Positive
*Thermoleophilum*	Aerobe	Negative
*TM7_genera_incertae_sedis*	Uncultured	
*Trueperella*	Anaerobe	Positive
*Varibaculum*	Anaerobe	Positive

**Table A4:** **Table listing all bacteria identified in this study that can be routinely cultivated and identified individually by standard methods described by the Health Protection Agency in routine investigations of urine (“Health Protection Agency, 2012”)**.

**Genus**	**Aerobe or Anaerobe**	**Gram status**
*Actinomyces*	Anaerobe	Positive
*Aerococcus*	Anaerobe	Positive
*Anaerococcus*	Anaerobe	Positive
*Arcanobacterium*	Anaerobe	Positive
*Arthrobacter*	Anaerobe	Positive
*Brevibacterium*	Aerobe	Positive
*Campylobacter*	Anaerobe	Negative
*Corynebacterium*	Anaerobe	Positive
*Enterobacter*	Anaerobe	Negative
*Enterococcus*	Anaerobe	Positive
*Eubacterium*	Anaerobe	Positive
*Finegoldia*	Anaerobe	Positive
*Fusobacterium*	Anaerobe	Negative
*Gardnerella*	Anaerobe	Variable
*Gemella*	Anaerobe	Positive
*Lactobacillus*	Anaerobe	Positive
*Mobiluncus*	Anaerobe	Positive
*Mycobacterium*	Aerobe	Positive
*Neisseria*	Aerobe	Negative
*Nocardioides*	Aerobe	Positive
*Paraprevotella*	Anaerobe	Negative
*Peptococcus*	Anaerobe	Positive
*Peptostreptococcus*	Anerobe	Positive
*Porphyromonas*	Anaerobe	Negative
*Prevotella*	Anaerobe	Negative
*Propionimicrobium*	Anaerobe	Positive
*Pseudomonas*	Aerobe	Negative
*Rhodococcus*	Aerobe	Positive
*Staphylococcus*	Anaerobe	Positive
*Stenotrophomonas*	Aerobe	Negative
*Streptococcus*	Anerobe	Positive
